# Conservative whole‐organ scaling contrasts with highly labile suborgan scaling differences among compound eyes of closely related *Formica* ants

**DOI:** 10.1002/ece3.2695

**Published:** 2017-01-24

**Authors:** Craig D. Perl, Sergio Rossoni, Jeremy E. Niven

**Affiliations:** ^1^ School of Life SciencesCentre for Computational Neuroscience and RoboticsUniversity of SussexFalmerBrightonUK; ^2^ Present address: Department of Physiology, Development and NeuroscienceUniversity of CambridgeDowning Site, Downing StreetCambridgeCB2 3EGUK

**Keywords:** evolutionary allometry, facet, static allometry, wood ant

## Abstract

Static allometries determine how organ size scales in relation to body mass. The extent to which these allometric relationships are free to evolve, and how they differ among closely related species, has been debated extensively and remains unclear; changes in intercept appear common, but changes in slope are far rarer. Here, we compare the scaling relationships that govern the structure of compound eyes of four closely related ant species from the genus *Formica*. Comparison among these species revealed changes in intercept but not slope in the allometric scaling relationships governing eye area, facet number, and mean facet diameter. Moreover, the scaling between facet diameter and number was conserved across all four species. In contrast, facet diameters from distinct regions of the compound eye differed in both intercept and slope within a single species and when comparing homologous regions among species. Thus, even when species are conservative in the scaling of whole organs, they can differ substantially in regional scaling within organs. This, at least partly, explains how species can produce organs that adhere to genus wide scaling relationships while still being able to invest differentially in particular regions of organs to produce specific features that match their ecology.

## 1 Introduction

Allometric scaling characterizes how organ size changes as organisms themselves increase in size (Huxley & Tessier, 1936). Typically allometric scaling relationships are power functions defined by two parameters: the intercept (*b)* and the power (α). Changes can occur in both intercept, referred to as grade shifts, and/or power, referred to as slope shifts. Scaling relationships can be classified in one of three principle ways (Cock, 1966; Gould, 1966): (1) ontogenetic allometry, which characterizes how an organ changes size as an organism develops (McLellan et al., 2002); (2) static allometry, which compares organ scaling among conspecifics at a given stage of development (typically adulthood; McCullough, Ledger, O'Brien, & Emlen, 2015); and (3) phylogenetic or evolutionary allometry, which compares the scaling of homologous/analogous structures between related species at a given taxonomic level (Voje, Hansen, Egset, Bolstad, & Pélabon, 2014).

The extent to which the intercept and/or the slope of an allometric relationship are evolvable traits has been heavily debated (Egset et al., 2012; Emlen & Nijhout, 2000; Mirth, Frankino, & Shingleton, 2016; Pélabon et al., 2014). Functional, developmental, or genetic constraints that restrict the morphospace in which organs have the potential to grow have been suggested to limit the extent to which allometries evolve (Bolstad et al., 2015; Pélabon et al., 2014). Pleiotropic effects have also been proposed to contribute to this limitation: changes in the mechanisms that generate allometry causing detrimental changes in other systems, thereby reducing overall fitness (Bolstad et al., 2015). Ontogenetic allometry has also been proposed to act as a developmental constraint limiting evolvability because evolutionary and static allometries are necessarily dependent on variability generated during development (Pélabon et al., 2014).

Despite these proposed limitations, however, there is substantial evidence showing that allometric scaling relationships can evolve (Emlen & Nijhout, 2000; Voje et al., 2014). This is supported by comparisons of static allometries that show they can differ within populations (Perl & Niven, 2016a), and among populations and species (Emlen & Nijhout, 2000; McGuigan, Nishimura, Currey, Hurwit, & Cresko, 2010; Simmons & Tomkins, 1996; Toju & Sota, 2006; Weber, 1990). Indeed, the idea that allometries can evolve is far from new: “…allometric trends are as subject to evolutionary alteration as are morphological features”. (Gould, 1966).

Grade shifts have been induced by artificial selection demonstrating that some aspects of allometric scaling can evolve rapidly (Bolstad et al., 2015; Frankino, Zwaan, Stern, & Brakefield, 2005, 2007). In contrast to the wealth of evidence demonstrating that intercepts can evolve, allometric slopes appear more constrained in their evolution, many organs showing remarkably little variation in scaling exponents between species separated by millions of years (Voje et al., 2014). Those experiments that have attempted to artificially select for slope shifts (Bolstad et al., 2015; Egset et al., 2012; Frankino et al., 2007; Stillwell, Shingleton, Dworkin, & Frankino, 2016; Tobler & Nijhout, 2010) have been criticized because of the methodology they employ (Mirth et al., 2016; Stillwell et al., 2016). Slope shifts induced by these experiments were often lost rapidly in subsequent generations once selection was eased (Bolstad et al., 2015), or were very minor changes (Stillwell et al., 2016; Voje et al., 2014).

Here, we investigate the evolutionary allometry of an organ by comparing the scaling of compound eyes in four species of ant from the genus *Formica*. We examine scaling of the entire compound eye through facet number, facet diameter, and eye area. Differences in scaling of facet diameter and facet number are indicative of relative changes in cell size and number, respectively (Chown et al., 2007; Montagne et al., 1999; Perl & Niven, 2016b). Both cell size and number contribute to changes in organ size, the differential contributions of facet number and facet diameter providing information about the mechanistic basis of changes in the size of a compound eye with increasing body size.

We also investigate regional differences within eyes through facet diameter scaling providing insight into how organs change size at a suborgan (cellular) level (Perl & Niven, 2016b; Stevenson, Hill, & Bryant, 1995). By measuring facet diameter scaling in different regions of the compound eye, we can also determine whether an overall change in eye size is produced by uniform changes across the whole eye, or through changes at different rates in different regions. Facet diameter scaling in *Formica rufa* differs among different regions of the compound eye (Perl & Niven, 2016b). By investigating these principles in related ant species, we examine not just the prevalence of evolutionary allometry among the genus but also the extent to which any differences in eye scaling between species can be explained through changes in intra‐eye scaling.

**Figure 1:**
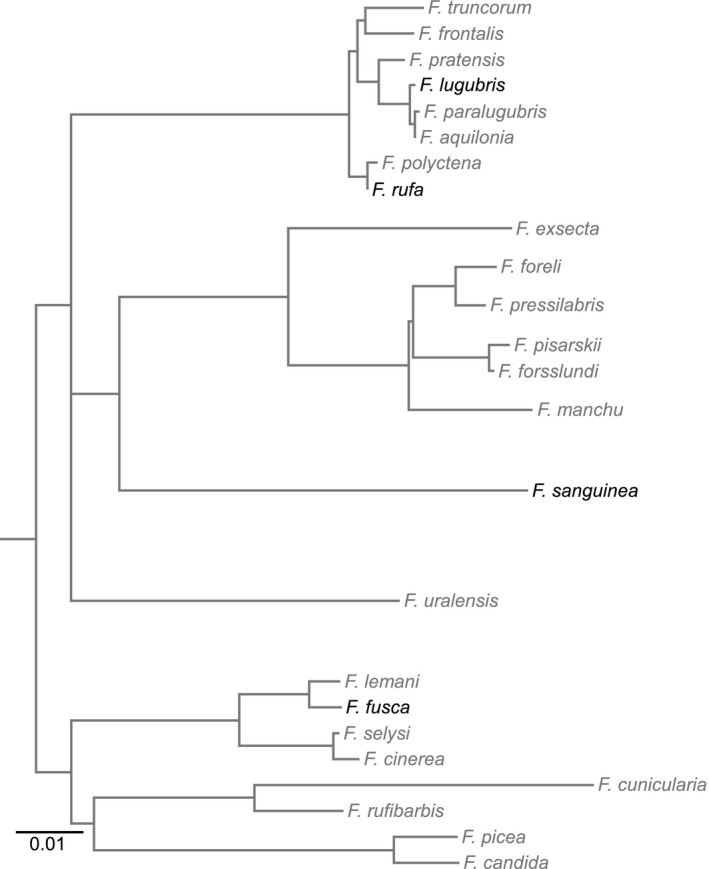
Phylogeny of *Formica* sp. Species used in this study are highlighted in black, whereas other species are in gray. Scale bar indicates nucleotide substitutions per site. Modified from Goropashnaya et al. (2012)

We selected ants based on their disparate phylogenetic positions (Figure 1, Goropashnaya, Fedorov, Seifert, & Pamilo, 2012) and ecologies. The most derived ants in our study are *F. rufa* and *Formica lugubris*, representing the clade *Formica sensu strictu*; both species build large, mound‐shaped nests in forested regions where they forage along trails for honeydew and invertebrate prey (Collingwood, 1979). In Britain, *F. lugubris* is polydomous, unlike the monodomous *F. rufa* (Collingwood, 1979). *Formica sanguinea* represent the Raptiformica; they are facultatively dulotic, raiding for slaves and freely foraging (Mori, Grasso, & Le Moli, 2000). *Formica fusca* are the most basal of the ant species we investigated, living in single‐ or double‐gyne nests of ~200 freely foraging workers (Collingwood, 1979; Wallis, 1964). Both *F. sanguinea* and *F. fusca* live in more open field or meadow habitats compared with the *Formica s. s*.

## 2 Materials and Methods

### 2.1 Animals

*Formica rufa* workers were collected from Ashdown Forest, UK (51.073, 0.043), between June 2013 and August 2014, whereas those of *F. fusca* were collected from University of Sussex campus, UK (50.864, −0.0800), in May 2014. Workers of *F. lugubris* were collected from forests on North Yorkshire Moors, UK (54.347, −0.883), in September 2014. *F. sanguinea* workers were collected from north of Cluj‐Napoca, Romania (46.862, 23.536), in August 2015. Table 1 shows the numbers of animals sampled.

**Table 1:** Number of ants and number of nests used per species for whole‐eye scaling and for intra‐eye scaling

	No. of nests	No. workers for whole‐eye scaling	No. workers for intra‐eye scaling
*Formica fusca*	2	34	34
*Formica lugubris*	3	52	23
*Formica sanguinea*	3	62	21
*Formica rufa*	3	63	65

### 2.2 Specimen preparation

Individual worker ants were selected from a colony at random and restrained with plasticine (Early Learning Centre, UK). Transparent nail varnish (Rimmel, London, UK) was applied to both compound eyes using a cocktail stick to create a cast (Ribi, Engels, & Engels, 1989). Ants were then stored at 4°C for a minimum of 48 hr to ensure the casts dried completely. These casts were removed, flattened, and mounted on to 12.5 mm specimen stubs (Agar Scientific, UK; Fig. S1). The eye casts and the left hind femur (as a proxy for body size) from *F. fusca*,* F. lugubris*,* F. rufa*,* and F. sanguinea* were mounted for subsequent measurement. Nail‐varnish eye casts and femurs were gold‐coated and imaged using a scanning electron microscope (S420 Stereoscan; LEO Electron Microscopy Ltd., Germany) or mounted on a microscope slide (Fig. S1) and imaged using a Zeiss Axioskop compound microscope (Carl Zeiss AG, Germany) and photographed using a micropublisher 5.0 RTV (Q‐imaging, Canada). Left hind femurs were imaged using a Leica MZ12.5 dissecting microscope (Leica, Germany) and photographed using a Canon EOS 7D SLR camera (Canon, Japan).

Sample sizes can be found in Table 1. The mean facet diameter per eye was obtained by measuring 36 facets per individual. Three facets were sampled from three different rows per eye region. The mean facet diameter for each eye region was then ascertained using the mean value of facet diameter from the facets in each specific region. The facet number was measured by counting every facet within an eye. The facet diameter was measured as the diameter of the facet along its longest axis. The eye area was measured by approximating the eye as an oval, which correlates almost exactly with the eye area measured directly (Perl & Niven, 2016a). Facet diameters, femur lengths, and facet numbers were all measured and counted from their respective micrographs or photographs using ImageJ (Schneider, Rasband, & Eliceiri, 2012).

### 2.3 Statistics

#### 2.3.1 Line fitting

There is significant debate in the literature concerning the most appropriate line‐fitting method for allometric data. Some authors, such as Stillwell et al. (2016), advocate using major axis or standardized (reduced) major axis regression (MA/SMA) on the basis that this accounts best for error in the method of fitting lines to allometric data. Other authors advocate using MA/SMA on the basis that this method accounts for error in the *x*‐ as well as the *y*‐axis (Warton, Wright, Falster, & Westoby, 2006). Additionally, MA/SMA removes assumptions concerning biological phenomenon being directly related (Stillwell et al., 2016). Major axis or standardized major axis regression lines should only be fitted when both *X* and *Y* variables are sampled randomly (Warton et al., 2006); however, we sampled a broad size range of ants to ensure appropriate coverage. The measurement error in our data is likely to be small compared with the (unavoidable) amount of equation error (i.e., data points not lying exactly on the regression line). It has been noted that estimating allometric slopes is inaccurate when there is substantial equation error (Egset et al., 2012). Therefore, we have selected ordinary least square regression to analyze our data, rather than MA/SMA.

#### 2.3.2 Statistical tests

Eye area, mean facet diameter, and facet number were analyzed using linear mixed‐effect models from the “nlme” package (Pinheiro, Bates, DebRob, Sarkar, & R‐core, 2016). Using the estimable function from the “gmodels” package (Warnes, Bolker, Lumley, & Johnson, 2015) and by constructing custom contrast matrices, we made *post hoc* multiple pairwise comparisons (*t* tests) of these linear mixed‐effect models to determine whether changes in slope and/or intercept had occurred. Nonsignificant model terms were eliminated stepwise until only significant terms remained in the model. All analyses were conducted with log‐transformed data to allow for valid interpretation of the allometric coefficients. Principle component analysis (PCA) and cluster analysis were conducted using the PCA and HCPC functions from the “FactoMineR” package, which uses agglomerative hierarchical clustering (Husson, Josse, & Pagès, 2010; Lê, Josse, & Husson, 2008).

In addition to gross morphological scaling, we investigated scaling in facet diameters from different regions of the compound eye. These data were also analyzed using linear mixed‐effect models with post hoc pairwise comparisons. All statistics were calculated using R v.3.1.2 (R Core Team, 2016), and all model structures can be found in Table 2.

**Table 2:** Structure of linear mixed‐effect models for all analyses. Individual ants were included as a random effect when they contributed more than one data point to a given model

Response	Fixed effect(s)	Random effect(s)
Mean facet diameter	Femur length	Nest
Facet number	Femur length + species	Nest
Eye area	Femur length + species	Nest
Facet number	Mean diameter	Nest
*Formica fusca* facet diameter	Femur length + eye region	Individual nested in nest
*Formica lugubris* facet diameter	Femur length + eye region	Individual nested in nest
*Formica sanguinea* facet diameter	Femur length × eye region	Individual nested in nest
*Formica rufa* facet diameter	Femur length × eye region	Individual nested in nest
Mean anterior facet diameter	Femur length × species	Nest
Mean dorsal facet diameter	Femur length + species	Nest
Mean posterior facet diameter	Femur length × species	Nest
Mean ventral facet diameter	Femur length × eye region	Nest

## 3 Results

### 3.1 Allometric scaling of compound eyes and facets of *Formica* species

We examined three aspects of the allometric scaling of the compound eyes of workers from four *Formica* ant species: (1) scaling of facet number, (2) scaling of mean facet diameter, and (3) scaling of eye area.

**Figure 2:**
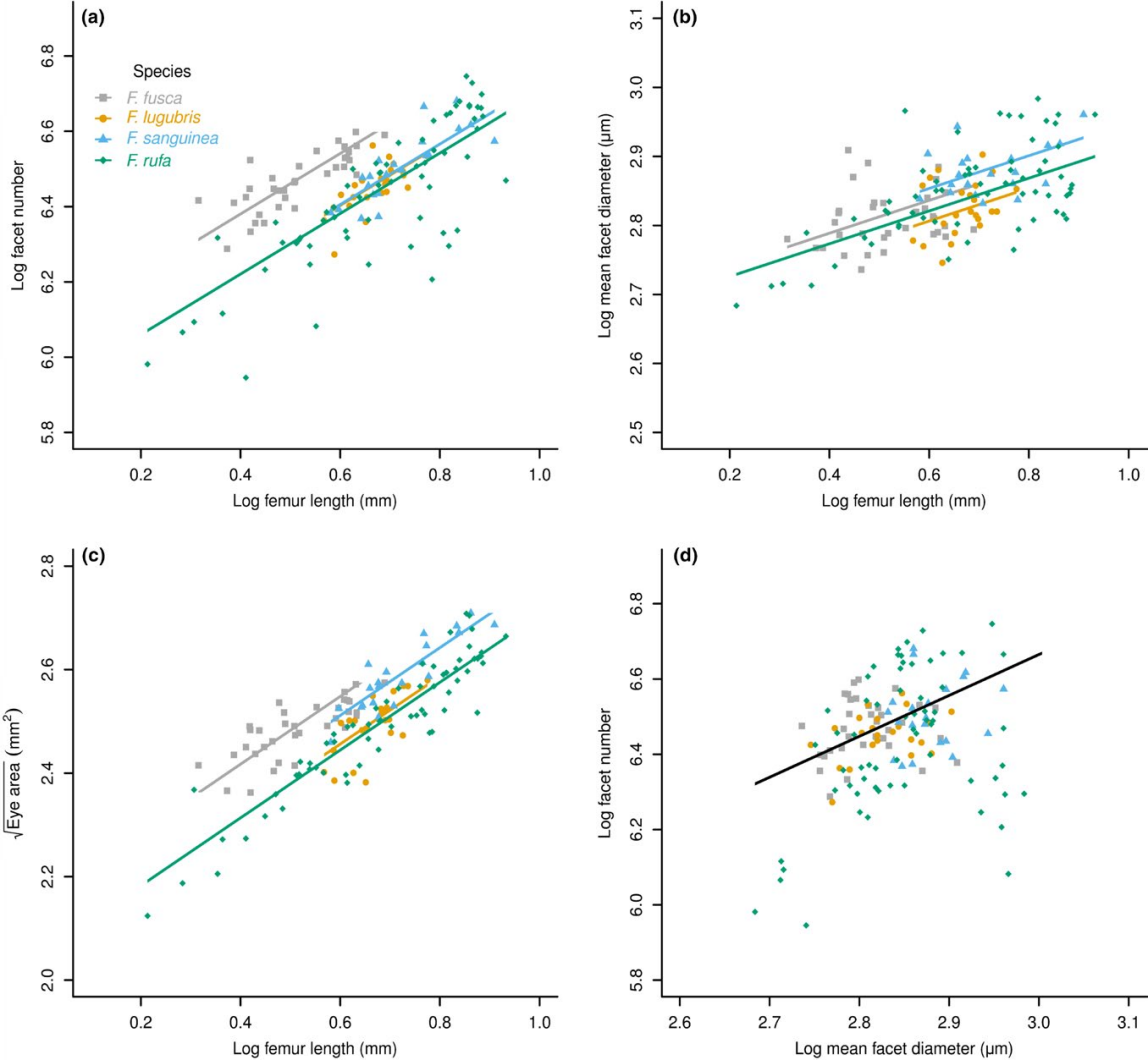
Scaling relationships in the four species of *Formica* as derived from linear mixed‐effect models. (a) Allometry of facet number per eye as a function of rear left femur length (a proxy of body size). (b) Allometry of mean facet diameter as a function of rear femur length. (c) Allometry of eye area as a function of rear femur length. (d) Scaling of mean facet diameter as a function of number of facets per eye among the four species of *Formica*. Colored lines are individual regressions for species, when significant differences exist among species. A single black regression line indicates no significant difference between species, which are analyzed with a common slope

Across the genus, facet number increased significantly with increasing hind femur length (*F*_141,128_ = 236.94, *p* < .001). The absence of a significant interaction between hind femur length and species (*F*_141,125_ = 0.31, *p* = .82) indicated that the slope (i.e., the rate of facet number increase with increasing femur length) did not differ across all four species (Figure 2a, S2a, Table S1). There was, however, a significant difference in the facet number among species (*F*_141,8_ = 4.85, *p* = .03), indicative of a grade shift (or a change in elevation). Pairwise comparisons revealed that facet number differed between *F. fusca* and the three other species: *F. lugubris* (*t*_141,8_ = 2.91, *p* = .02); *F. rufa* (*t*_141,8_ = 3.67, *p* < .01); and *F. sanguinea* (*t*_141,8_ = 2.88 *p* = .02). There were no differences between the other species pairs (*t*_141,8_ < 0.60, *p* > .57). Therefore, for a given body size, *F. fusca* workers have more facets than do workers of the other three species. Despite this difference, the rate of increase in facet number with body size was the same across all four species. Facet number scaled with a negative allometry for all four species, α < 1 (Table 3), indicating that larger ants had relatively fewer facets than smaller ants.

**Table 3:** Scaling exponents ± standard error for each species for each scaling relationship

	*Formica lugubris*		*Formica sanguinea*		*Formica rufa*		*Formica fusca*	
	α	logβ	α	logβ	α	logβ	α	logβ
Facet count versus femur length	0.85 ± 0.19	5.88 ± 0.13	0.87 ± 0.13	5.88 ± 0.10	0.90 ± 0.08	5.82 ± 0.06	0.66 ± 0.09	6.13 ± 0.05
Mean facet diameter versus femur length	0.24 ± 0.15	2.67 ± 0.10	0.16 ± 0.08	2.76 ± 0.06	0.26 ± 0.04	2.67 ± 0.03	0.09 ± 0.08	2.77 ± 0.04
Eye area versus femur length	0.78 ± 0.16	1.97 ± 0.11	0.69 ± 0.08	2.09 ± 0.06	0.69 ± 0.03	2.02 ± 0.02	0.45 ± 0.07	2.25 ± 0.03

Slope = α, intercept = β. Scaling exponents were extracting from linear models (see Supporting information), to maintain consistency with other allometric investigations. Supplemental graphs (Figs S2–S4) show slope and intercept estimates from linear models.

Mean facet diameter also increased significantly with increasing hind femur length across the genus (*F*_141,128_ = 73.86, *p* < .001). There was no significant interaction term between hind femur length and species (*F*_141,125_ = 0.21, *p* = .89), and, therefore, the slope did not differ across all four species (Figures 2b, S2b, Table S1). There was also no significant difference in mean facet diameter between species (*F*_141,8_ = .21, *p* = .89). Thus, there were no slope or grade shifts between any of the species. The rate of facet diameter increase is the same across workers of all species as is the mean facet diameter for a given size of worker. Mean facet diameter scaled with a negative allometry across all four species, α < 1 (Table 3), indicating that larger ants had relatively smaller facets than their smaller counterparts.

As expected from the previous analyses, the square root of eye area (used to preserve dimensionality among different response variables) increased significantly with increasing hind femur length across the genus (*F*_141,128_ = 646.08, *p* < .001). Again, there was no significant interaction term in the model (*F*_141,128_ = 0.66, *p* = .58), indicating that the slope did not differ across all four species (Figures 2c, S2c, Table S1). There was a significant difference in mean eye area (*F*_141,8_ = 8.74, *p* < .01) indicative of a grade shift: *F. fusca* differed from both *F. lugubris* (*t*_141,8_ = 3.67, *p* < .01) and *F. rufa* (*t*_141,8_ = 4.50, *p* < .01); *F. sanguinea* also differed from both *F. lugubris* (*t*_141,8_ = 2.37, *p* < .05) and *F. rufa* (*t*_141,8_ = 3.18, *p* = .01). There were no further differences between the species (*t*_141,8_ < 1.47, *p* > .18). Thus, *F. rufa* and *F. lugubris* have a similar eye area for a given body size, as do *F sanguinea and F. fusca*. However, although the rate of increase in eye area with increasing body size is similar across all species sampled, *F. fusca* and *F. sanguinea* have a larger area compound eye for a given body size compared with members of *Formica s. s*. Eye area scaled with a negative allometry across all four species, α < 1 (Table 3), indicating that larger ants have a relatively smaller area eyes than their smaller counterparts.

### 3.2 Scaling of facet number with diameter

By assessing the scaling of facet diameter with facet number, we were able to assess their relative contributions to the overall structure of the compound eye. Facet number increased significantly with increasing mean facet diameter across the genus (*F*_141,128_ = 17.61, *p* < .001). There was no significant interaction term in the model, indicating that the slope did not differ across all four species (*F*_141,125_ = 0.61, *p* = .61; Figures 2d, S2d, Table S1). There were also no significant differences among all four species (*F*_141,8_ = 0.12, *p* = .95), indicating that there were no shifts in intercept. Thus, the rate of facet diameter increase with increasing facet number is similar across all the species sampled. Likewise, the mean facet diameter for a given number of facets is the same across all species sampled.

**Figure 3:**
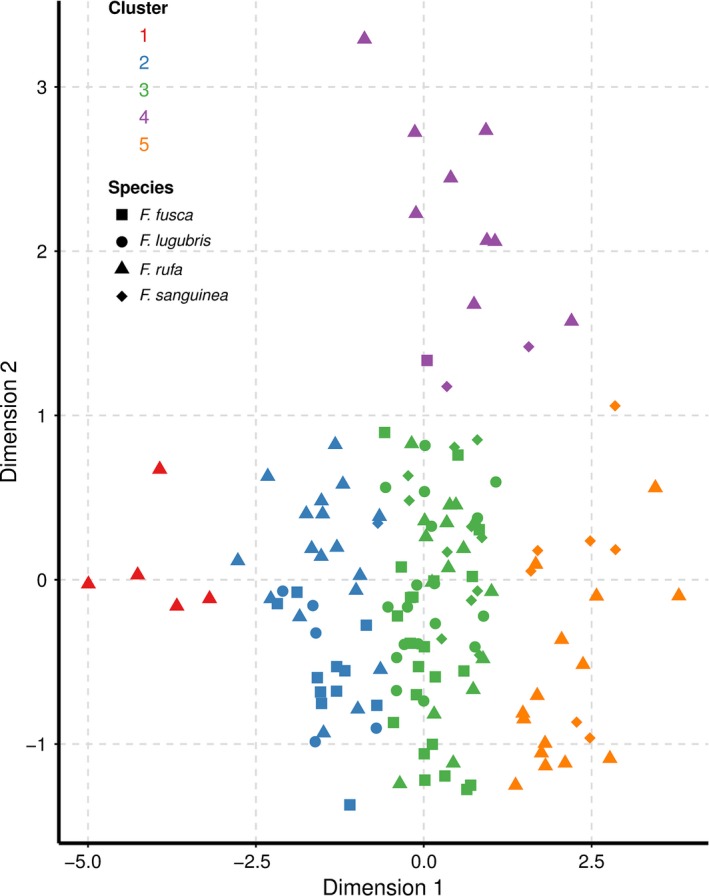
Hierarchical cluster analysis of facet number, mean facet diameter, and species after dimension reduction using principle component analysis (PCA). Clusters are defined with different colors, while different species are represented with different shapes

We assessed the differences in facet number and facet diameter with the overall area of the compound eye among the four species using PCA followed by cluster analysis (see Section 2, Figure 3, Table 4). We used the PCA to reduce the three variables of interest (facet number, mean facet diameter, and eye area) to two principle components. The first two principle components explained 97.4% of the variation in the data: Dimension 1 was strongly positively correlated with eye area, while dimension 2 was moderately positively correlated with facet count and moderately negatively correlated with facet diameter. Subsequent agglomerative hierarchical cluster analysis revealed that there were five clusters (Figure 3). Only one cluster consisted of a single species, *F. rufa*. Indeed, *F. rufa* appeared in all five clusters, more than any of the other species (Figure 3). The remaining clusters were all formed from at least two species, with two clusters having representatives from all four species.

**Table 4:** Correlations, eigenvalues, and relative contributions of all three factors for all three principle components

	Principle component 1		Principle component 2		Principle component 3	
	Correlation	Contribution	Correlation	Contribution	Correlation	Contribution
Count	0.84	32.45	−0.52	36.14	0.16	31.41
Diameter	0.72	23.86	0.69	63.37	0.10	12.78
Area	0.98	43.70	−0.06	0.49	−0.21	55.81
Eigenvalues						
Variance	2.181		0.741		0.077	
% of variation	72.72		24.71		2.58	

### 3.3 Intra‐eye scaling

#### 3.3.1 Within species

We next examined the scaling of facet diameter in different regions (Fig. S1) of the compound eye in each of the four species. For each species, we determined the allometric scaling of facet diameter in the anterior, dorsal, posterior, and ventral regions of the compound eye.

**Figure 4:**
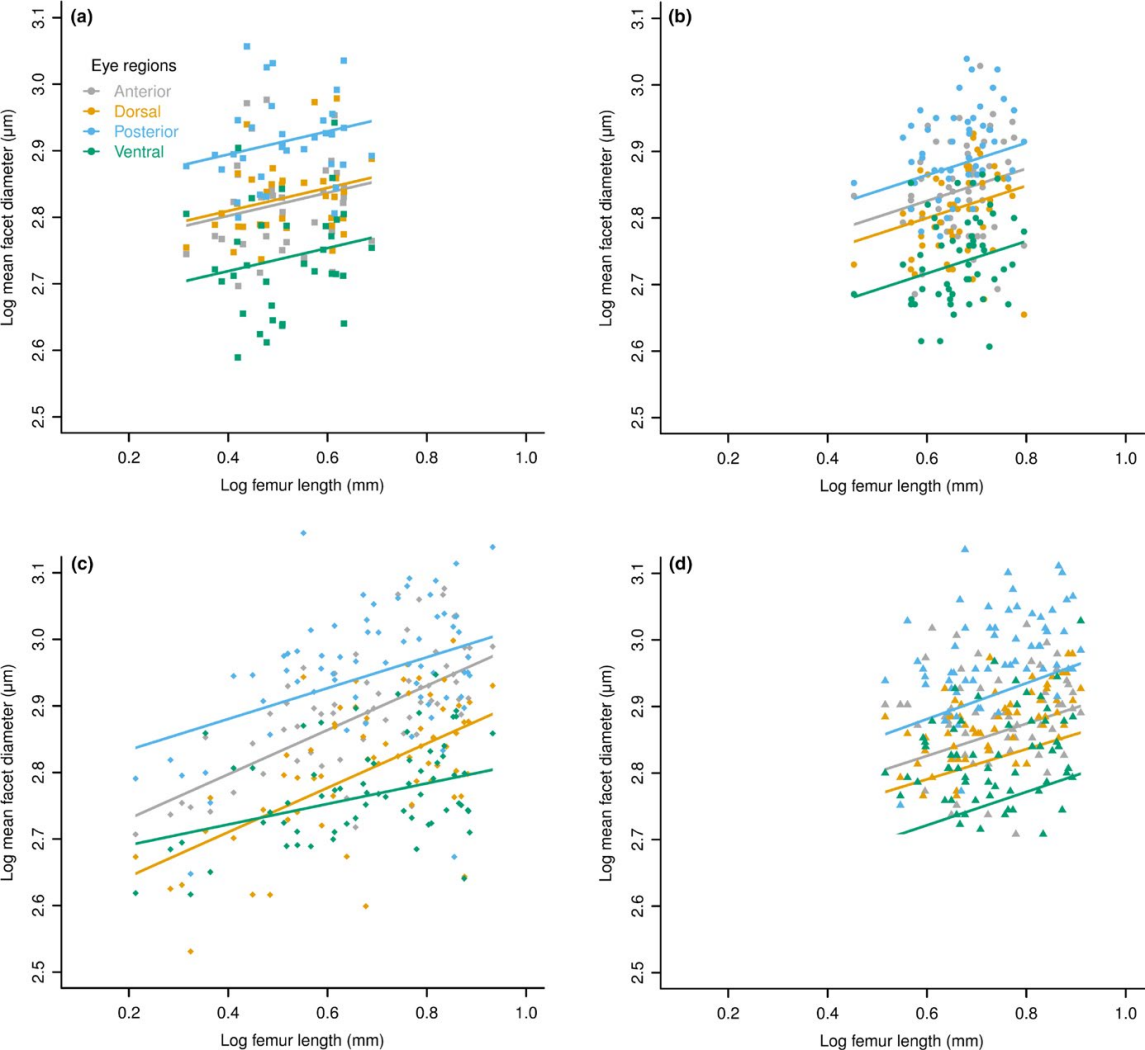
Intra‐eye facet diameter scaling within species as derived from linear mixed‐effect models. Comparison of the scaling of mean facet diameters in different regions of the compound eyes from (a) *Formica fusca*; (b) *Formica lugubris*; (c) *Formica sanguinea*; (d) *Formica rufa*

There were differences in the scaling shifts that occurred within the eyes of different species. Within the *F. fusca* compound eye, there were no slope shifts (*F*_34,96_ = 0.11, *p* = .95), indicating that the rate of mean facet diameter increase with increasing body size is the same in each region of the eye. The facet diameters in different regions showed grade shifts relative to one another *F*_34,99_ = 39.52, *p* < .0001; Figures 4a, S3a, Table S2). Aside from anterior and dorsal regions (*t*_34,99_ = 0.45, *p* = .65), all other regions were grade‐shifted relative to each other (*t*_34,99_ > 5.15, *p* < .0001). Thus, for a given body size, the anterior and dorsal regions have similar mean facet diameters, with the posterior facets being the larger and ventral facets being the smallest.

Facet diameters in different regions of the *F. lugubris* compound eye also did not exhibit any slope shifts (*F*_52,150_ = 0.02, *p* > .99), only showing grade shifts (*F*_52,153_ = 65.41, *p* < .0001). Unlike *F. fusca*, all regions were significantly different from each other (*t*_52,153_ > 3.07, *p* < .01; Figures 4b, S3b, Table S2). Again, the rate of mean facet diameter increase with increasing body size is similar across all regions of the eye. The posterior region facets are the largest for a given body size, followed by anterior and dorsal with ventral facets being the smallest.

Comparisons among the facet diameters from the four regions in *F. rufa* showed both slope (*F*_65,188_ = 4.00, *p* < .01) and grade shifts within the eye (*F*_65,188_ = 116.743, *p* < .0001). There were grade shifts between the posterior and all other eye regions: anterior (*t*_65,188_ = 2.88, *p* = .004), ventral (*t*_65,188_ = 2.98, *p *< .01), and dorsal (*t*_65,188_ = 4.92, *p* < .0001). (Figures 4c, S3c, Table S2). There were no differences between the intercepts of the other pairs (*t*_65,188_ < 1.47, *p* > .1). The facet diameters in the anterior (*t*_65,188_ = 2.90, *p* < .01) were slope‐shifted relative to the ventral region. There were no further slope shifts (*t*_65,188_ < 1.94, *p* > .05). Thus, the mean diameter of facets in the posterior region is larger than those in the anterior, ventral, and dorsal regions. The rate of facet diameter increase with increasing body size is faster in the anterior regions of the eye than in the ventral region.

Although there was no significant interaction term for the model (*F*_62,180_ = 2.30, *p* = .08), pairwise comparisons between the different regions of the *F. sanguinea* compound eye showed a significant slope shift between facet diameters in the anterior and posterior regions of the eye (*t*_62,180_ = 2.38, *p* < .02; Figures 4d, S3d, Table S2). There were no further slope shifts between regions (*t*_62,180_ < 1.85, *p* > .07) nor were there any grade shifts (*t*_62,180_ < 1.93, *p* > .05). Facet diameter scaling is, therefore, similar among all regions of the eye, except between the anterior and posterior regions: The mean facet diameters in the posterior region increase at a greater rate with body size compared with those in the anterior region.

#### 3.3.2 Among homologous regions from the compound eyes of different species

**Figure 5:**
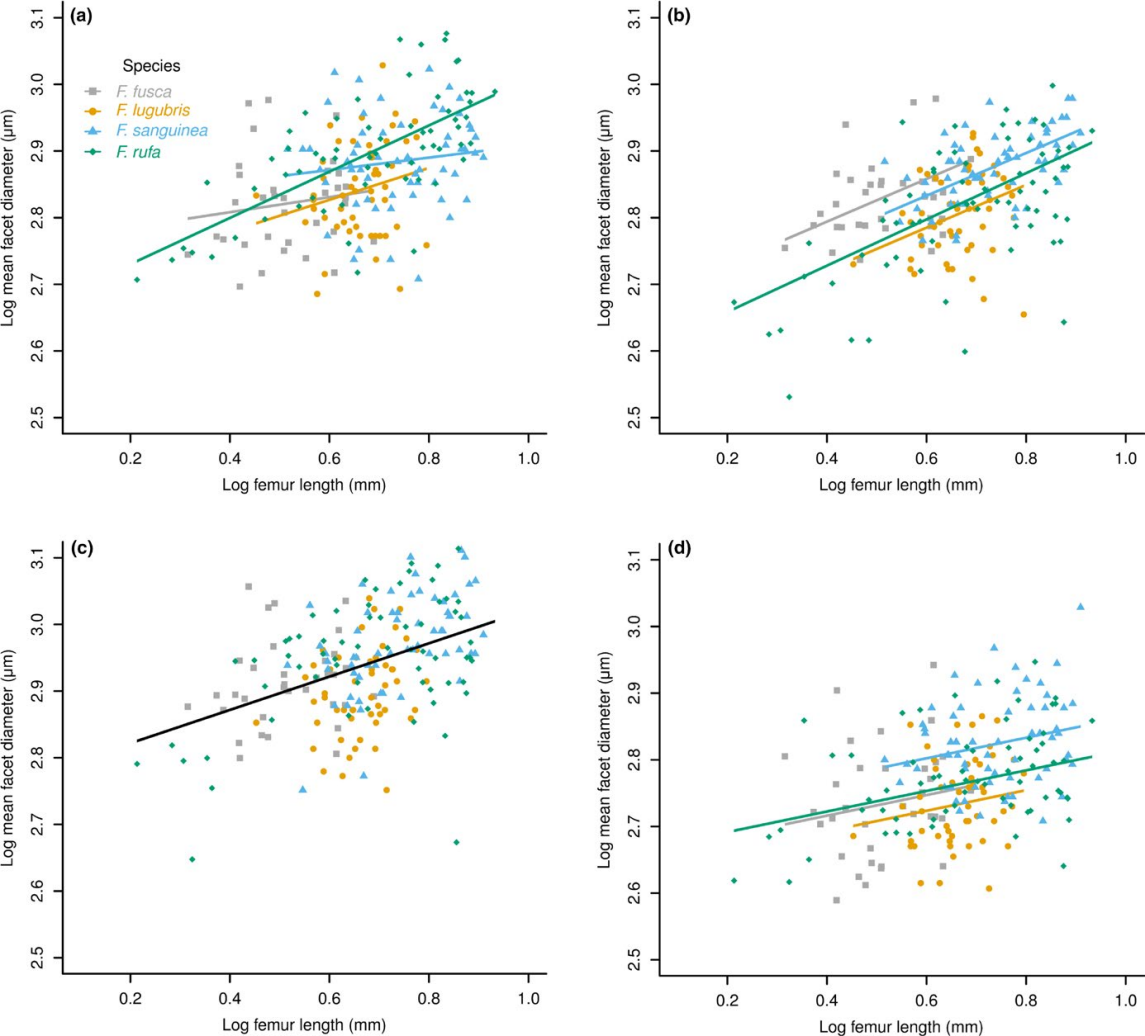
Intra‐eye facet diameter scaling among species as derived from linear mixed‐effect models. Comparison of the scaling of mean facet diameters from homologous regions of the compound eyes of the four *Formica* species. Mean facet diameter scaling of (a) the anterior region; (b) the dorsal region; (c) the posterior region; (d) the ventral region. Colored lines are individual regressions for species, when significant differences exist among species. A single black regression line indicates no significant difference between species, which are analyzed with a common slope

Homologous eye regions (Figure 2) scaled differently among the four species. In the anterior region of the eye, there was a significant slope shift among different species (Figures 5a, S4a, Table S3). Although there was no significant interaction term for the model (*F*_213,197_ = 2.57, *p* = .055), pairwise comparisons revealed a significant grade shift between the mean anterior facet diameters of *F. rufa* and *F. sanguinea* (*t*_213,198_ = 2.47, *p* < .01). There were no significant differences between the slopes of other species (*t*_213,7_ < 2.05, *p* > .07). Likewise, there were no grade shifts between the facets of the anterior region between any of the species (*F*_213,7_ = 3.63, *p* = .07). As body size increases, facet diameters in the anterior of the *F. rufa* compound eye increase faster than those of *F. sanguinea*.

There was no significant slope shift in facet diameters from the dorsal region of the compound eye across different species (*F*_213,198_ = 0.41, *p* = .75), but there were significant grade shifts (*F*_213,7_ = 5.84, *p* = .03; Figures 5b, S4b, Table S3). The mean dorsal facet diameters were grade‐shifted between *F. rufa* and *F. fusca* (*t*_213,7_ = 3.34, *p* = .01) as well as between *F. rufa* and *F. sanguinea* (*t*_213,7_ = 2.76, *p* < .03). There were further grade shifts between *F. lugubris* and both *F. fusca* (*t*_213,7_ = 3.12, *p* = .02) and *F. sanguinea* (*t*_213,7_ = 2.40 *p* < .05). There were no further grade shifts between the dorsal regions of any of the other species (*t*_213,7_ < 1.06, *p* > .32). *Formica fusca* have a larger mean facet diameter in the dorsal region of the compound eye than *F. rufa* for a given body size. The diameters of the dorsal facets of *F. sanguinea* are also larger than those of *F. rufa* for a given body size*. Formica lugubris* have smaller dorsal fact diameters than either *F. fusca* or *F. sanguinea*.

There was no significant slope shift in the posterior region of the eye across different species (*F*_213,198_ = 0.48, *p* = .69) nor were there any significant grade shifts (*F*_213,7_ = 0.95, *p* > .47; Figures 5c, S4c, Table S3). Consequently, there were no differences between species in terms of either mean facet diameter for a given body size or in the rate of facet diameter increase with body size in the posterior regions of the eye.

As with the dorsal and posterior regions of the compound eye, there was no significant slope shift in the ventral region of the eye across different species (*F*_213,198_ = 0.38, *p* = .77) but there were significant grade shifts (*F*_213,201_ = 15.23 *p* < .0001; Figures 5d, S4d, Table S3). The mean ventral facet diameters were grade‐shifted between *F. sanguinea* and *F. lugubris* (*t*_213,7_ = 3.16, *p* = .02). There were no further grade shifts in ventral facet diameters between any of the other species (*t*_213,7_ < 1.89, *p* > .10). The mean diameter of facets in the ventral region of eye of *F. sanguinea* is larger for a given body size than that from *F. lugubris*.

## 4 Discussion

By comparing the static allometric scaling relationships governing compound eye size, facet number, and diameter across closely related species, our findings demonstrate that evolutionary shifts exist in the allometric scaling of organs. At the whole‐eye level, changes in static allometric scaling relationships are restricted to grade shifts, with slope shifts entirely absent. This supports previous claims based on comparisons among closely related species or artificial selection experiments that allometric scaling relationships can evolve but that grade shifts are easier to achieve than slope shifts (Bolstad et al., 2015; Emlen & Nijhout, 2000; Frankino et al., 2005, 2007; Pélabon et al., 2014; Tobler & Nijhout, 2010; Toju & Sota, 2006; Voje et al., 2014).

Despite grade shifts in the allometric scaling of the compound eye among the four *Formica* species in terms of eye scaling, the scaling of mean facet diameter with facet number is remarkably consistent; no grade or slope shifts occurred among the species. This is supported by the PCA/cluster analysis in which workers do not cluster based entirely on their species. The high degree of conservation of the relationship between mean facet diameter and facet count may indicate that developmentally or functionally related traits are not necessarily as free to vary as those same traits are with body size. Under artificial selection, Frankino et al. (2005, 2007) demonstrated that a genetically and functionally linked trait (hind‐wing size of a butterfly) can be forced into alternative scaling regimes, indicating that the restrictions on functionally linked morphological traits are not necessarily developmental/genetic (Mirth et al., 2016). Pélabon et al. (2013) also concluded that constraints on evolutionary allometry are the consequence of selection, rather than due to a developmental limitation. If this is the case for the relationship between facet diameter and facet number in the present study, it implies that the relationship is maintained through selection across the genus and that deviating from this reduces fitness.

Changes in scaling across the entire organ are not the only way in which changes can occur in static allometric scaling relationships, and they can also occur at the suborgan level (Perl & Niven, 2016b). In contrast to the relatively conservative changes in the allometric scaling relationships of the whole compound eye among the four *Formica* species, we found substantial variability in the allometric scaling relationships of facets in specific regions of the compound eye. Both grade shifts and slope shifts occur among regions. The patterns of facet diameter scaling between eye regions appear unique to each species, as well as to any particular region among species. As such, these differences could explain species‐specific adaptations while adhering to genus wide relationships at the level of the entire eye.

The intra‐eye differences are mediated primarily through grade shifts so that for a given body size, facet diameter depends upon the eye region in which that facet resides, but the rate at which facet diameter increases is the same across the different eye regions. However, slope shifts occur between one or more regions in two species: *F. rufa* and *F. sanguinea*. This demonstrates that the way in which evolutionary changes occur in static allometries is far more nuanced than implied by mean measurements sampled from across the entire organ.

To expand upon this further, the scaling of eye area is consistent between different species of the genus. However, the means by which they all arrive at the same scaling rules does differ. There is no difference in mean facet diameter scaling between species. However, intra‐eye facet diameter scaling differs vastly depending on the region the facets are in, and the species to which they belong. Grade shifts in facet number scaling also occur between some species. Therefore, consistency in eye area scaling is maintained through differential scaling of facet diameters and relative investment in facet number. A given eye area can be obtained through either changes in facet number or diameter. Thus, two eyes may have the same area, one composed of large numbers of small facets and the other of fewer, larger facets. Among our four species, some may change the scaling in the anterior portion of their eye relative to the other regions, whereas another may scale the posterior region instead. This is combined with grade shifts in facet number. Through this mechanism, the scaling of eye area is the same across the genus, while individual species display differential facet diameter scaling in different regions of the eye.

The differences in intra‐eye scaling between species are further emphasized when examining scaling shifts between homologous regions of different species. In two of the four regions investigated (ventral and dorsal), at least one species pair demonstrated grade shifts, although the patterns of grade shifts were different between regions. Between the dorsal region of different species, there ample grade shifts with only *F. fusca* and *F. sanguinea* being similar along with *F. lugubris* and *F. rufa*. In contrast, anterior facet diameters show only slope shifts, but only between a single pair of species: *F. rufa* and *F. sanguinea*. This implies that allometric shifts across evolutionary timescales are not simple changes that affect entire organs or even parts of organs in the same way. Thus, even though slope shifts did not occur between species when looking at scaling at a whole‐organ level, slope shifts do occur between homologous regions within the compound eyes of different species. Furthermore, grade shifts that are not apparent when examining whole‐organ allometry become obvious when examining within‐organ scaling.

Slope shifts are purportedly less common than grade shifts in evolutionary allometry (Egset et al., 2012) and difficult to maintain across generations even when induced through strong artificial selection (Bolstad et al., 2015; Stillwell et al., 2016). However, our analysis demonstrates that slope shifts do occur, even between closely related species, although not at a whole‐organ level. Thus, species with different life histories and foraging habits have similar investment in mean facet diameter as a function of facet number but differ in facet diameter scaling relationships between the homologous eye regions. This implies that the internal proportions of an organ are far freer to vary than the rate of organ size increase with body size, explaining how compound eyes can be specifically adapted to particular visual ecologies while conforming to specific scaling relations at a genuswide level.

Although our findings demonstrate changes in the static allometric scaling among and within the compound eyes of closely related species, there is a lack of phylogenetic consistency in these scaling relationships. Moreover, allometric shifts do not appear to be related to life history or ecology irrespective of whether they are at the whole‐eye or intra‐eye level. This may be a consequence of the relatively sparse sampling of species or a lack of sufficiently detailed descriptions of the visual ecologies of the species we studied. Detailed studies of the behavior, physiology, and morphology of single species have shown that eye regionalization of this sort is very common in insects (Land, 1997) and that it is often associated with specific behavioral requirements that provide a strong selective incentive, such as mate (Collett & Land, 1975; Kirschfeld & Wenk, 1976) or prey detection (Labhart & Nilsson, 1995). Even though we cannot attribute regional changes in facet diameter to specific behavioral and ecological requirements, our results show that not only do regions scaling differentially within a species (Perl & Niven, 2016b) but that closely related species can evolve substantial differences in homologous regions.

## Conflict of Interest

None declared.
