# Supplementary material for: Conservative whole‐organ scaling contrasts with highly labile suborgan scaling differences among compound eyes of closely related Formica ants
Source: Ecol Evol. 2017 Jan 24;7(6):1663–73. doi: 10.1002/ece3.2695 (PMC5355196; doi:10.1002/ece3.2695)
Supplement: Supplementary file 5 [file ECE3-7-1663-s005.docx]

**Supplemental data for:**

**Conservative whole-organ scaling contrasts with highly labile sub-organ scaling differences among compound eyes of closely-related *Formica* ants**

**Craig D. Perl, Sergio Rossoni, and Jeremy E. Niven**

This supplement details the eye casts used in the study and demonstrates the homology between eye regions (Figure S1). It also contains figures S2-S4, which show the same data as in the main text but with the regression lines plotted using the estimates from linear models rather than the linear mixed effect models. These figures indicate the allometric relationships without accounting for nest affiliation. The estimates of these slopes and intercepts (allometric exponents) can be found in Table 3 of the main text.

*Figure legends*

**Figure S1.** Eye casts from the four species used in this study. (A) A representative electron micrograph of an eye cast from *Formica fusca.* (B) A representative photo an eye case from *F. lugubris.* (C) A representative electron micrograph of an eye cast from *F. rufa.* (D) A representative photo an eye cast from *F. sanguinea.* Scale bars = 100µm. A,D,V,P refer to anterior, dorsal, ventral and posterior, respectively. Visible smudges on the casts of *Formica lugubris* (B) and *F. rufa* (D) are artefacts caused by casts having been removed from SEM stubs and re-mounted to microscope slides for the preparation of this figure.

**Figure S2.** Allometric scaling relationships in the four species of *Formica* as derived from linear models. (A) Allometry of facet number per eye as a function of rear left femur length (a proxy of body size). (B) Allometry of mean facet diameter as a function of rear femur length. (C) Allometry of eye area as a function of rear femur length. (D) Scaling of mean facet diameter as a function of number of facets per eye among the four species of *Formica*.

**Figure S3.** Intra-eye facet diameter allometric scaling within species as derived from linear models. Comparison of the allometric scaling of mean facet diameters in different regions of the compound eyes from: (A) *Formica fusca;* (B) *F. lugubris*; (C) *F. sanguinea*; (D) *F. rufa*.

**Figure S4.** Intra-eye facet diameter allometric scaling among species as derived from linear models. Comparison of the allometric scaling of mean facet diameters from homologous regions of the compound eyes of the four *Formica* species. Mean facet diameter scaling of: (A) the anterior region; (B) the dorsal region; (C) the posterior region; (D) the ventral region.

**Table S1.** Slope and intercept estimates derived from linear mixed effect models analysing differences between *Formica* sp. in terms of whole organ eye scaling.

|  | Estimate | ± SE |
| --- | --- | --- |
| Facet count vs femur length | | |
| Slope | 0.80 | 0.05 |
| *F. fusca* intercept | 6.06 | 0.04 |
| *F. lugubris* intercept | 5.92 | 0.05 |
| *F. rufa* intercept | 5.90 | 0.04 |
| *F. sanguinea* intercept | 5.92 | 0.05 |
|  |  |  |
| Mean facet diameter vs femur length | | |
| Slope | 0.24 | 0.03 |
| *F. fusca* intercept | 2.69 | 0.02 |
| *F. lugubris* intercept | 2.66 | 0.03 |
| *F. rufa* intercept | 2.68 | 0.02 |
| *F. sanguinea* intercept | 2.71 | 0.03 |
|  |  |  |
| Eye area vs femur length | | |
| Slope | 0.65 | 0.03 |
| *F. fusca* intercept | 2.16 | 0.02 |
| *F. lugubris* intercept | 2.06 | 0.02 |
| *F. rufa* intercept | 2.05 | 0.02 |
| *F. sanguinea* intercept | 2.12 | 0.03 |
|  |  |  |
| Facet number vs mean facet diameter | | |
| Slope | 1.09 | 0.20 |
| Intercept | 3.41 | 0.56 |

**Table S2.** Slope and intercept estimates derived from linear mixed effect models analysing differences in facet diameter scaling between different regions of the eye within different *Formica* sp.

|  | Estimate | ± SE |
| --- | --- | --- |
| *F. fusca* |  |  |
| Slope intercept | 0.17 | 0.08 |
| Anterior intercept | 2.73 | 0.04 |
| Dorsal intercept | 2.74 | 0.04 |
| Posterior intercept | 2.83 | 0.04 |
| Ventral intercept | 2.65 | 0.04 |
|  |  |  |
| *F. lugubris* |  |  |
| Slope | 0.24 | 0.14 |
| Anterior | 2.68 | 0.09 |
| Dorsal | 2.65 | 0.09 |
| Posterior | 2.72 | 0.09 |
| Ventral | 2.57 | 0.09 |
|  |  |  |
| *F. sanguinea* |  |  |
| Anterior intercept | 2.68 | 0.09 |
| Dorsal intercept | 2.65 | 0.09 |
| Posterior intercept | 2.72 | 0.09 |
| Ventral intercept | 2.57 | 0.09 |
| Anterior slope | 0.24 | 0.14 |
| Dorsal slope | 0.23 | 0.18 |
| Posterior slope | 0.27 | 0.18 |
| Ventral slope | 0.25 | 0.18 |
|  |  |  |
| *F. rufa* |  |  |
| Anterior intercept | 2.66 | 0.04 |
| Dorsal intercept | 2.58 | 0.04 |
| Posterior intercept | 2.79 | 0.04 |
| Ventral intercept | 2.66 | 0.04 |
| Anterior slope | 0.33 | 0.05 |
| Dorsal slope | 0.33 | 0.06 |
| Posterior slope | 0.23 | 0.06 |
| Ventral slope | 0.15 | 0.06 |

|  | Estimate | ± SE |
| --- | --- | --- |
| Ventral region |  |  |
| Slope | 0.15 | 0.04 |
| *F. fusca* intercept | 2.65 | 0.03 |
| *F. lugubris* intercept | 2.63 | 0.03 |
| *F. rufa* intercept | 2.66 | 0.03 |
| *F. sanguinea* intercept | 2.71 | 0.03 |
|  |  |  |
|  |  |  |
| Posterior region |  |  |
| Slope | 0.25 | 0.04 |
| Intercept | 2.77 | 0.03 |
|  |  |  |
| Anterior region |  |  |
| *F. fusca* intercept | 2.76 | 0.07 |
| *F. lugubris* intercept | 2.68 | 0.10 |
| *F. rufa* intercept | 2.66 | 0.03 |
| *F. sanguinea* intercept | 2.82 | 0.07 |
| *F. fusca* slope | 0.11 | 0.13 |
| *F. lugubris* slope | 0.24 | 0.20 |
| *F. rufa* slope | 0.35 | 0.14 |
| *F. sanguinea* slope | 0.09 | 0.16 |
|  |  |  |
| Dorsal region |  |  |
| Slope | 0.32 | 0.04 |
| *F. fusca* intercept | 2.67 | 0.03 |
| *F. lugubris* intercept | 2.59 | 0.03 |
| *F. rufa* intercept | 2.59 | 0.03 |
| *F. sanguinea* intercept | 2.64 | 0.03 |

**Table S3.** Slope and intercept estimates derived from linear mixed effect models analysing differences in facet diameter scaling within homologous regions of the eye between different *Formica* sp.
